# Transactions of the Medical Society of the County of Kings

**Published:** 1865-06

**Authors:** 


BUFFALO

### Medical and Surgical Journal.

VOL. IV. JUNE, 1865. No. 11.

### ART. I.—*Transactions of the Medical Society of the County of Kings.*

REGULAR MEETING, FEBRUARY, 1864.

### *Puerperal Convulsions.*

DR. J. E. CLARK reported a case of puerperal convulsions in a woman, aged 30, the mother of three children, and now eight months' pregnant. He saw the patient on Wednesday. She was suffering from intense headache, for which leeches were applied to the temples. Next day headache continued; urine albuminous, and swelling about the face. Seen in consultation with Dr. McClellan, and cupped over region of kidneys. Did well until Sunday, when she became much worse. Dr. McClellan being out, Dr. Enos was called. During the consultation she had a convulsion. Chloroform was administered. The os was dilated and the woman delivered of a living child. The convulsions then subsided. The patient had three convulsions between Sunday and Monday morning. On Thursday convulsions came on again, and continued until death, which occurred on Saturday. Patient died comatose. Dr. Enos made a post mortem examination. The skin was of a bright yellow color. As the patient had not been seen for twelve hours before death, it could not be determined whether this was an ante-mortem or post-mortem appearance. The lungs were healthy; heart fatty; liver fawn color, enlarged, and very fatty; kidneys were enlarged and fatty.

### *Paralysis.*

DR. JOHNSON reported a case of paralysis of the right arm, after whooping-cough, in a child 2½ years old. The paralysis occurred after a severe spasm. The arm is wasted to one-half the size of the other. He had another case following scarlet fever. After playing by the window on a cold day, the child complained of pain about the cervical vertebræ. The next day there was complete paralysis of both feet and arms. Treated by counter irritation along the course of spine. Child has recovered the use of both arms and partial use of legs. The paralysis yielded in the right arm first.

*Imperforate Anus.*

Dr. Johnson also reported a case of imperforate anus. The child had not been examined as carefully as it should have been at birth, and was supposed to be properly developed; but having no passage from the bowels the Doctor was called, and found imperforate anus. Nothing, but a fissure, where the anus should be. After waiting five or six days in order to allow the distention of the gut to indicate the most expedient point, vomiting occurred, and on the seventh day he operated, penetrating to the depth of an inch and a quarter, at which point he reached the rectum. The wound was kept open by the daily introduction of the finger. This he did himself for two weeks and then entrusted it to a nurse. From the neglect of the nurse the wound closed, and the Doctor was called again, to find the operation a failure. He operated again, and this time continued the dilatation for one month. The child is now well.

Dr. Minor stated in relation to this case, that he had operated a few years ago upon a child for imperforate anus, in which case there was a stellate appearance in place of the anus. He operated on the third day after the birth of the child; cut up about one inch, using curved bistory, and guiding the knife by the concavity of the sacrum. The wound was afterwards kept open by the introduction of a finger stall of very thin India rubber. The finger stall was introduced first, and then filled with wool until the anus was dilated to the size desired. The result was very favorable. The child recovered with power to retain fæces.. Dr. M. thought this method of dilating the wound very preferable to the one used by Dr. Johnson. He had attempted the introduction of a sponge tent, but found the pain and irritation too severe to continue it.

Dr. Minor had operated in another case where he went up two and a half inches, and failing to find the gut the operation was discontinued.

*Varicocele.*

Dr. Minor related the treatment of a case of varicocele in a young man, on the left side. He had at first used a suspensory bandage, but with little relief. The plan he now adopted was to thrust the testicle well up, and bandage the lower portion of the scrotum with adhesive plaster to retain the testicle in position. The patient is doing well, and has a fair prospect of rcovery.

*Spinal Meningitis.*

Dr. Minor also briefly reported a case of spinal meningitis, arising from dentition. The most prominent symptoms were opisthotonos and great prostration. The patient recovered under the use of blisters, tonics and electricity.

*Anomalous cases of Parturition and Gestation.*

Dr. I. T. Conkling related the following case: He was called to a case of labor in a primipara, in which the head presented in the right occipito-posterior position. The occiput corresponding with the right sacro-iliac symphysis and the os frontis with the left acetabulum. Instead of rotating to the left anterior, and the occiput emerging beneath the pubis, the occiput passed into the hollow of the sacrum and becoming arrested the forceps were applied, and the child removed with face anteriorly.

This presentation is regarded by Naëgelé as the second in frequency, yet the non-rotation of the head is very rare. The rotation occurring in all but 27 of 1254 cases recorded by Naëgelè. In all of 26 cases mentioned by Staltz, in all but 2 cases of 76 by Barry, in all but 39 of 303 by Dubois.

Dr. Hart thought such cases particularly adapted to the use of the forceps, and remarked that in no case ought a child to be marked by the skillful application of the forceps.

Er. Norris (coroner) stated that he had been recently called upon to hold an inquest upon a child whose frontal bone and those of the face had been crushed by the forceps.

Dr. Housel stated that he had recently been called in consultation by *Mrs.* Dr. Redmond. The patient, a woman aged 35, had had severe labor pains for thirteen hours, but which had entirely ceased three hours previously. Patient was conscious; pulse weak; virtex at the superior strait. He advised the use of the forceps, which was refused, lest it might injure the reputation of attending physician. Two hours after head had receded. He decided there was rupture of the uterus. Dr. Byrne was then called as additional counsel. Dr. B. introduced his hand into the uterus, found laceration upon the posterior surface of the uterus. He found no pulsation in the cord, and proceeded to deliver by the feet. The head could not be removed; applied forceps and failed; patient sinking. An incision was then made in the neck, and the blunt hook attached to the forceps attached to the occipital bone and head delivered. Woman died in four hours. No post mortem.

Dr. Norris exhibited a specimen of certificate from a mid-wife, in which she had forged the name of a physician. He gave his views as to certificates of death, and thought they should be received only from regular practicing physicians.

Dr. Conkling stated that he was called in January last to see a woman who was eight months' pregnant with her sixth child, on account of the edematous condition of her lower limbs. Otherwise felt well. No albumen in urine. But slight relief was obtained from the swollen condition of the extremities, and she gave birth to her child at the expected time by a short, easy, and natural labor. The child cried faintly; the cord was enormously distended with fluid; the body was completely anasarcous, and purplish, and its face so much swollen as to nearly obliterate its features. No pulsations could at any time be detected in the radial arteries, and its respirations, which were at first scarcely perceptible, ceased ten hours after birth. Post mortem eighteen hours after death. No fluid found in the abdomen or pleural cavities, but the pericardium was distended with water so that by its mechanical obstruction it must have caused death.

Dr. Hart regarded this case of hydrops peracardii as an exceedingly rare one.

Dr. Norris mentioned the case of a young girl who died very suddenly. The peracardium was found distended with water—the cause of death.

Dr. Norris also stated that the case of the pugilist, Bill Poole, was somewhat parallel. He was shot, the ball entering the septum between the ventricles, and became encysted there. The aperture through which it entered healed, but the inflammatory process resulted in throwing out slowly, for six weeks, a quantity of water in the cavity of the perieardium, sufficient to impede the heart's action and cause death. At the post mortem examination everything was found healed, but an accidental cut of the knife struck the encysted ball,

Dr, Ball mentioned a case of phlebitis occurring during gestation, in order to elicit the views of members in regard to the propriety of producing premature labor in cases where the life and future health of the mother are involved.

In July last was called to see a lady, aged 43 years, who was suffering from some constitutional disturbance, attended with suppression of the menses, constipation, etc. Thinking that it must be the change of life with her, from her age, and the fact that she had not conceived for twenty years, he paid but little attention to the suppression, but prescribed for the more general symptoms. In August he saw her again, and found her suffering very severely from phlebitis of the left lower extremity, which she said had troubled her since her last confinement.

Upon a thorough investigation of the case he satisfied himself of the existence of pregnancy, when he ordered her perfect rest, and prescribed for the difficulty, which was much relieved for awhile, but after a few weeks it returned, and involved both limbs and extended up into the iliac regions, causing extreme pain and restlessness.

The disease continued to increase in severity, and the patient becoming so much exhausted from the loss of sleep and from suffering as to imperil her life. He was considering the propriety of interference when nature came to his relief by bringing on premature labor in the eighth month of gestation. He thought it an important question as to what circumstances would justify the practitioner in superceding nature in such cases.

### REGULAR MEETING, MARCH, 1864.

### *Poisoning by Oil of Tansy.*

Dr. Norris reported the following case:

A short time ago a case of poisoning by the oil of tansy came under his notice. The accused, on his examination stated, that he had administered only *ten* drops of this article, but the probability is, that he had given a great deal more. At all events, the woman, soon after swallowing the potion, was seized with convulsions, fell in the street, was taken home, and expired soon afterwards, in one of the paroxysms. On opening the body the odor of tansy was very perceptible, and an analysis of the contents of the stomach, afterwards, revealed the fact of its presence most conclusively. It seemed, in fact, to permeate the whole system, the mucous membrane of the stomach and duodenum being soft and intensely inflamed. On the right side was found a minute gland, with a very small duct, which afterwards proved to be the kidney; but it was evidently arrested in its development, and was of no service whatever. The specimen was presented at the last meeting of the Pathological Society of New York, and elicited a great deal of discussion. The left kidney was large, and had to perform all the duty. The vessels of tbe brain were engorged with blood, and fluid was found in the ventricles.

On opening the uterus, it was found to be impregnated; the embryo could be clearly seen, and the communication of the placenta distinctly traced. A great point of interest in the case, was the abnornal kidney.

The question here recurred, what was the cause of death, and in what way did the tansy produce this sudden and fatal result? In his opinion it produced a poisoning of the blood, and prostration of the nervous system. The question, however, has never been satisfactorily determined, and no one had yet clearly determined how this oil causes death. Tansy has been known to have caused fatal results in ℥ss doses, but he did not think that ten drops would have that effect. He was aware that this article was used to procure abortion, but he did not believe it was a *specific* in these cases.

Dr. Ball thought it a very intricate question, to determine the cause of death in poisoning from the oil of tansy; but imagined that it produced a poisoning of the blood, and as a consequence prostration of the *nervous* system.

Dr. Gardner knew of one case where tansy *did* produce abortion. A woman, quite young, and the mother of a family, complained while on a visit in the country last August, that she was pregnant, and desired very much to get rid of the *fœtus.* The same evening a strong decoction of tansy was given to her; in the morning a hemorrhage, so profuse, occurred, that a doctor had to be sent for. The fœtus was finally expelled, and her own life, with difficulty, saved. She again came under his care in January, suffering from rheumatism, and general debility; and while under treatment, informed him that she had been pregnant for the last three months. A profuse hemorrhage soon afterwards occurred, and she had to assume the recumbent position; administered ergot, and was told next day that the fœtus had come away. Such, however, was not the case; it was merely a *uterine mole*, a portion, most likely, of the placenta, left behind since last summer.

Dr. Gardner was sent for last summer, to attend another lady who had frequented the skating ponds, and received many severe falls. She was not aware of being in the family-way. When he arrived at the house, she had profuse uterine hemorrhage, and a little while afterwards an ovum came away. The falls, in this instance, may have produced loss of vitality in the embryo, and the ovum remained behind for some time before being expelled.

Dr. Ball desired to know what was meant by a "false conception;" whether it was a proper term to make use of, and whether it was the result of previous *abortions?*

Dr. Norris was not averse to the term, and thought it meant a "blighted ovum." He attended a lady, some time ago, who supposed herself pregnant for three or four months. She was flooding pretty freely, and he administered ergot. Soon afterwards she passed off a shapeless mass of flesh, resembling a placenta, and about the size of the fist. This lady had had no previous miscarriages. Thought that this mass was a blighted ovum.

Dr. Gardner related a case where a uterine "mole" was thrown off some months subsequent to a miscarriage.

Dr. Hart was cognizant of several instances, where ladies threw off these false conceptions. One lady, in particular, had four attacks of the kind before a child was born. She afterwards had several children. He could also remember another case, where the lady threw off two shapeless masses, and had never been pregnant previously. She afterwards had children.

Dr. Gardner stated, that some time ago, he was called to assist a physician in a case of arm presentation. Introduced the hand with great difficulty into the uterus, turned and delivered a good sized child. On examination found another child in utero; the pains soon came on, and she was delivered of the second. The placenta was single, but on the membranes of the second child a rudimentary placenta was attached without cord.

*Local Experiences.*

During the last month Dr. Hart had seen a great deal of measles, where the cough was severe, respiration quick, and afterwards congestion of the lungs with typhoid diathesis. The rash seemed to be more or less livid in color.

Dr. Gardner stated that he had seen several instances of scarlatina with diphtheria of a malignant type. Was called one morning to see a child with this disease, and found the skin covered with the rash, the neck very much swollen, and breathing very much impeded; she died the same evening. A child of the same family was taken sick shortly afterwards, with the same complaint, where the cellular tissue of the neck was enormously swollen, although the child could swallow pretty well. Thought that an abscess was forming, and found that by the advice of some friends, a large poultice had been applied. The parts suppurated, and soon after the breaking of the abscess the child died, probably from syncope.

Dr. Ball had two or three cases of measles occurring during whooping cough. In these cases the whooping cough seemed to moderate during the febrile excitement, but the measles pursued its regular course, although the eruption was scanty. He had also seen several cases of billious fever, during the last month.

Dr. Hart mentioned a case of membranous croup, which terminated fatally.

*Puerperal Convulsions.*

Dr. Gardner had attended a woman three weeks ago in a very severe attack of puerperal convulsions. It was her first child, and three days previous to her confinement complained of feeling very unwell; the bowels were very costive, and the feet swoolen. When labor set in, examined and found a head presentation, but noticed that the membranes did not come down. The labor was tedious, and about 8½ P. M. she suddenly gave a scream, and was seized with violent convulsions. Sent immediately for assistance, and Dr. Burge soon afterwards arrived with the forceps. Chloroform was administered, but in consequence of rigidity of the soft parts, could not apply the instruments properly. The membranes, however, soon protruded, and were ruptured, producing a loud noise. The pains succeeded each other rapidly; the head came down, and the soft parts projected enormously. By a careful manipulation these were pushed back over the head, and no laceration took place in delivery. The patient was very uneasy all this time, and it was 12½ A. M. before the child was born. Mechanical means had to be resorted to to resuscitate the child, but it died very suddenly the next day. The convulsions continued for twenty-eight hours at intervals of twenty minutes to one hour; they then subsided, and she is now in a fair way to recover. The urine was limpid; but could not examine it satisfactorily, as it came away involuntarily, and was mixed with the other discharges. Did not think the convulsions were owing to uremic poison, but caused by the great sluggishness of the bowels, and the woman's own carelessness in not taking any cathartic medicines. Dr. Gardner stated that he had also blistered the neck very freely, and for a few days had to resort to beef tea injections to support the system.

Dr. Norris saw a similar case about five years ago, where he bled freely from both arms, and thought it the only means of controlling the convulsions.

Dr. Hart said that he had been very fortonate in his obstetrical practice, having never met with any formidable cases of puerperal convulsions.

Dr. Ball had attended several pretty severe cases of the kind, and had bled freely. He thought it a very proper and judicious treatment.

### *Baker Brown's Operation for Lacerated Perineum.*

Dr. Johnson related a case of Baker Brown's operation for lacerated perineum, in which he assisted Dr. Farley. The case was one of instrumental delivery, and the laceration extended through the recto-vaginal fourchette to the rectum. The operation was performed on the eighth or ninth day after delivery. The sutures were part superficial, and part through the whole mass of tissue, and these latter were tied on each side of the laceration over pieces of a gum elastic bougie cut off for the purpose. The elasticity of these served to tighten the sutures and keep the edges of the wound in perfect coaptation. The operation was performed with facility, and resulted without accident in union by the first intention.

Dr. Johnson next called for the experience of the members present in the treatment of prolapsus of the vagina, and elicited from Drs. Landon and Enos a description of Dr. Emmet's operation, which consists in abrading bands of the prolapsed wall in such a manner as to infold by sutures a culdesac of sound membrane which ultimately contracts, hardens and disappears. The operation may be repeated as often as necessary to restore the prolapsed wall to its normal length.

### *Atalectasis.*

Dr. Enos reported the case and post mortem of a child nineeent months old, to which he was called the day before its death. It had never been healthy, and its appearance at the time he saw it, indicated some malformation of the heart or large blood vessels. It was breathing 80 times per minute, and livid. On post mortem the foramen ovala was found open, and the posterior part of the left lung in a state of complete atalectasis, evidently having never been expanded.

### *Erysipelas.*

Dr. Bell reported a case of erysipelas of the ear, characterized by extensive swelling and severe pain. He at first applied to the inflamed surface, his favorite remedy, a glycerine solution of iodine. But to the surrounding temporal region, which was extremely painful, he ordered the application of the tincture of the root of aconite. This was followed by such relief as to suggest its further application to the erysipelatous surface, from which time, on the fourth day after disease, and when it was at its worst, the aconite was substituted in full strength, every two or three hours, for the iodine, and gave the patient great relief. Internally three grains of quinine and five minims of muriated tincture of iron were given every three hours, with a generous diet. Yet convalescence has been slow, and the blood evidently in a bad state, as the patient has had pupuric spots over the whole cutaneous surface.

### *Inhalation in Diphtheria.*

Dr. Bell also reported a case of diphtheria of a young lady, aged 21, and previous good health. February 27, after two or three days' "sore throat," she was suddenly seized with a violent dyspnœa and almost incessant cough. In this condition he first saw her. A pail of hot water was quickly prepared for the inhalation of watery vapor. This was followed by temporary relief, but after frequent applications during the day, ceased to give any alleviation. Diphtheritic membrane was apparent on the inferior border of the tonsils and on the back of the throat, but by no means in such quantity as to present an obstacle to respiration. The respiratory murmers were on inspiration sibilant, as in asthma; but the expiratory were fine, moist and weak. Believing the paroxysms of dyspnœa to be wholly due to a spasmodic action of the muscles of the larynx, (probably excited however by the presence of diphtheritic deposit,) appropriate anti-spasmodics were clearly indicated, and inhalation appeared to be the best means of application. To this end fifteen drops of the fluid extract of hemlock were added to about an ounce and a half of warm water in an inhaling tube an inch in diameter, and, with some difficulty at first, on account of the spasmodic action of the larynx, the operation commenced. The patient persisted, however, and in about twenty minutes was relieved. She soon afterwards fell into a quiet sleep, which lasted three hours. Twice subsequently during the night, (it was first used about 9 o'clock P. M.,) the remedy was repeated with the same result. But on awaking the third time, about five o'clock next morning, while she was threatened with another paroxysm she complained of severe temporal headache and dryness of the throat, and was in a copious cold sweat.

This condition being attributable to the hemlock, it was discontinued, and, in its place the tincture of musk was substituted, using at first twenty-five drops, but subsequently a teaspoonful ro the ounce and a half of water. This acted equally well, and without unpleasant consequences. Being obliged to be absent for the afternoon, Dr. Kissam was called upon and requested to attend in the case in emergency. About five o'clock P. M. another paroxysm came on so suddenly and violently, that the patient could not hold the tube. In the strait Dr. Kissam very promptly but carefully used chloroform without producing insensibility, with relief. During the following night and the next day the musk inhalation was used at the *very beginning of the paroxysm*, with the effect of subduing it five times. On the fourth day twice, and not afterwards.

Other treatment consisted of, during the first day, a syrup of ipecac emetic, with an apparent aggravation of the spasm, an aperiant aided by an assafœtida injection; and, during the whole course of the disease, two and a half grains of quinine with five drops of the muriated tincture of iron every three hours, whisky and beef tea, all she could be induced to take, which was but little. During the first three days the membrane increased about the fauces and downwards out of sight. On the fourth day it softened, and from this time was as usual offensive, breaking down and thrown off—the patient with a slight cough hawking up a mouthful at a time. She is now fairly convalescent, taking the quinine and iron three times a day, and generous diet.

The case elicited an informal discussion on the utility of inhalation, and the general prevalence of diphtheria at the present time. Dr. Enos, in particular, mentioned several cases, and one which he thought would have been an appropriate case for treatment by inhalation, other remedies having failed. All appreciated alike, however, the usually fatal issue of such cases, especially in children, in whom it was manifest that inhalation in the manner described, could not be used.

### *Fatty Degeneration of the Heart and Kidneys.*

Dr. Conkling presented a heart and kidneys, (which had undergone fatty degeneration,) and a uterus from the same patient, who died eighteen hours after delivery. From the commencement of gestation she suffered from excessive *nausea* and *vomiting*. When he first saw her, then in the seventh month of pregnancy, she was greatly debilitated, being unable to walk across the room. The feet and limbs were greatly swollen; the face swollen and pallid; pulse 130; respiration rapid; stomach rejecting all kinds of nourishment, and hemorrhage from the nose occurring daily. No albumen was found in the urine. Efforts were made to allay the vomiting, and support the system, but she did not improve in strength. As her life was in danger, if allowed to complete the natural term of pregnancy, the question of interfering to induce premature labor, was presented to the family, as the less of two evils, though the induction of labor might cause fatal hemorrhage from the uterine sinuses, as in her anermic state, the blood was thin and watery. Dr. Enos saw her, in consultation, February 6th, 1864, and advised the injection of warm water into the vagina, two or three times daily. This was continued until Friday, February 12th, when slight pains were felt through the hips, and on the 13th labor pains came on, and she was delivered of a living child, the breech presenting. The uterus contracted firmly, and but little blood was lost. The following morning she was feeble, but bore the free use of nourishment and stimulants without vomiting. She rallied, however, but little, and afterwards continued to lose strength, and died on the night of the 15th.

At the post mortem, which was made by Dr. Speir, the heart was found small, and covered with fat, its muscular fibres undergoing the process of fatty degeneration. The kidneys were fatty. The liver was also fatty and greatly enlarged.

### *Condyloma.*

Dr. Willson presented a specimen of condylomatous growth of the labia minora, which he had removed the day previously by the *ecrasseur*. The disease was clearly traceable to syphilitic origin, and had attained about twice the size of a fig. The patient was put under the influence of chloroform, and the parts then removed. The operation lasted about ten minutes, and was attended with very trifling hemorrhage. This patient was also suffering from an obstinate stricture of the rectum, which had existed about six months, and which had lately rendered defecation impossible, except the bowels were kept soluble by the use of cathartices or injections. At a previous examination he had been unable to pass the index finger into the rectum. In this contingency he availed himself of the advice and assistance of Dr. Hutchison, who recommended the forcible dilatation of the rectum, and suggested the use of a common kid glove stretcher to accomplish that object. After the operation, heretofore mentioned, he attempted to insert the point of the stretcher, but failed to do so. Dr. Hutchison, then, by main force, passed his index finger its whole length into the rectum; the stretcher was then inserted without difficulty three or four inches, and by moderate force the gut dilated to nearly, if not quite, its natural size. A slight discharge of blood succeeded the operation. The diminution in the calibre of the rectum was found to have been caused by thickening of its walls, occasioned probably by the same disease that existed in the vulva. As an additional obstacle to the passage of the *fæces*, there was a band of condyglomatous growth, seemingly stretching from opposite sides of the gut. This band was broken down. The stricture did not extend more than three inches above the anus. As a justification of the operation, it may be stated that the patient was relieved in a few moments of a very distressing and inconvenient complaint. It is possible that the relief afforded may be only temporary, but it is hoped that by the judicious use of *bougies*, a permanent cure may be effected.

Dr. Hutchison presented a specimen of rupture of the rectum, occurring in a female 40 years of age, under the care of Dr. Parker of New York. The patient had been suffering for a long time from stricture of the rectum, and about two weeks ago he was asked by the attending surgeon to assist in an operation for its removal. On examination the finger could be passed up the rectum for two or three inches, but not being long enough, it could not pass beyond the stricture. The patient being placed under the influence of chloroform, a *bougie* was passed very carefully up the rectum. As soon as it reached the stricture, the patient made a violent expulsive effort, and the bougie, as we supposed, passed through the obstruction. The operation was finished, and both left with the understanding that he was to attend for the time being. About half an hour afterwards was sent for in great haste, and on his arrival found the patient suffering from intense pain and vomiting, which continued during the night, and until death ensued next morning.

A post mortem was held thirty hours after death. The rectum was found ruptured, and the fæces had passed into the peritoneal sac, exciting peritonitis. Supposed the rupture occurred at the time of the operation; when the patient made the violent expulsive effort, the bougie passed through the *gut*, which was very thin at the seat of stricture, and not through the *obstruction*. Did not ascertain much of the history of the case, except that for the last two years the fæcal discharges had been growing smaller and smaller, until at length they became about the size of a No. 9 catheter. The specimen was examined under the microscope by Dr. Speir, but he could not detect any of the ordinary characteristics of cancerous disease. Still he would not like to say positively that it was not malignant.

Dr. Minor was cognizant of a similar case of stricture, in an aged person, which was supposed to be malignant. The patient went to Philadelphia, and was under the care of Dr. Pancoast, who introduced an instrument into the rectum, and dilated the stricture. The gentleman returned home soon afterwards, and the improvement in his condition was so manifest as to put every precedent at fault.

In reply to a question, Dr. Hutchison stated that he did not know whether any tubercular trouble existed in the lungs, as they had not been examined. It was with great difficulty that a post mortem could be obtained, and hence the examination was confined to the abdominal cavity alone.

If the stricture was not malignant in character, it must have commenced as an inflammation, followed by ulceration, producing a cicatrix, which gradually closed up the rectum. A softening of the part, at the seat of the stricture, was very apparent, and he could pull away with facility, loose portions of the tissue.

Dr. Enos remarked, that upon examining the specimen, he noticed that the stricture was *anteriorly*, and not at the curve of the sacrum. He thought, therefore, that the bougie could not have been forcibly pushed in.

DR. MINOR alluded briefly to the means used for dilating strictures, and that a safer and better mode of treatment might be adopted in cases similar to the one spoken of. He had operated on a child for *imperforate anus*, and used a small *India rubber finger stall;* he introduced it in a collapsed state into the intestine, after the incision was made, and then filled it with sheep's wool, until a sufficient dilation was effected. The softness of the rubber, and the elasticity of the wool acted admirably, and the child finally recovered. He thought the introduction of bougies, in strictures of the rectum, was not alwas a safe operation, and accidents were liable to occur. These, however, could not happen, were a method similar to the one be used in the imperforate anus, resorted to.

DR. A. OTTERSON thought that Dr. Minor was very fortunate in his operation for imperforate anus. Three years ago he operated on a child for this trouble, but could not succeed in keeping the gut open for more than seven months, at the end of which time the child died.

In regard to strictures of the rectum he would remark, that he had a specimen in his office, where there was softening around the stricture, the patient having died suddenly from a rupture of the bowels.

### *Intra-Pericardial Cyst.*

DR. ENOS presented a specimen of intra-pericardial cyst, mitral insufficiency, adherent pericardium, and hypertrophy, a sequence of rheumatism. It was taken from a delicate girl, twenty years of age, who five years ago had articular rheumatism, in the progress of which she had endocarditis, accompanied by a *bellows murmur*, most intense at the apex, indicating mitral regurgitation. Her feet became swollen, and she suffered from *dyspnœa.* She improved, however, so that the swelling disappeared, and she had tolerable health, until three weeks since. Her breathing, however, was always short, and the *bruit* did not pass away.

When called to see her three weeks since, she was pale; her countenance *anemic*, and somewhat anxious; the breathing hurried, and she was unable to lie down. The heart was much enlarged, its action rapid, but regular. There was a mitral regurgitant murmur, which could be traced along the spine, from the lumbar region to the summit of the head. The lungs were resonant, but there was a loud râle over both lungs, and a rattling sound, like that of parchment compressed. There was pain in the left side. The *bowels* were torpid, the *urine* normal, and the *menses* absent for months. The feet began to swell again, and some hemorrhagic spots were seen on the insteps. Respiration became more hurried, and death finally ensued.

On post mortem found the left *pleura* adherent in the lower part; both lungs were pushed aside by the heart, whose weight was thirty ounces, with the pericardium, which was adherent by not very strong adhesions. A *cyst*, about the size of a butternut, was formed, apparently of this effused material. It was in the right auriculo-ventricular groove, and contained a clear serum. All the *valves* were normal, except the mitral, which was contracted and insufficient. The *left auricle* was greatly expanded, and the *appendix* so dilated as to readily admit the finger.

The Doctor remarked that the great *hypertrophy* was occasioned by the valvular disease, and the adherent pericardium; that the *cyst*, which was the first he had ever met with in this location, was doubtless rubbed up, and elaborated out of the effused plastic material, by the motions of the heart, very much as a *bursa* is formed, to aviod friction between two gliding surfaces.

*Spurious Pregnancy; Its Symptoms, diagnosis and treatment, with a Record of Cases; Paper by* Dr. E. N. Chapman.

Nine cases of spurious pregnancy of a marked character having fallen under my observation, I purpose to give their histories, and venture a few remarks explanatory of the phenomena they presented. Doubtless most of the members of the Society have met with similar instances; and I hope, by a comparison of observations and opinions, we may arrive at a probable, or at least a plausible, explanation of the causation of this morbid condition, which simulates gestation so exactly as often to deceive the most experienced accoucheurs. A delusion of this kind may readily hold possession of the woman's mind in the earlier months, when a medical man even would be unable, satisfactorily, to clear up the doubts in the case, since he has nothing but sympathetic disturbances, aptly called signs, to guide him; but it is somewhat remarkable that this pseudo-pregnancy may advance step by step through all the stages of a genuine one, and apparent labor pains set in, when lo! on examination, the uterus is found of its natural size, and the conception and attendant manifestations a *lusus naturæ*.

Case I.—M. S., aged 42, the mother of twelve children and the subject of one miscarriage, applied at the clinique May 1, 1863, for the purpose of having it determined whether or not she was in the family-way. She supposed herself pregnant from the fact that her courses, unless interrupted by gestation or lactation, had never failed since their first appearance, excepting at two periods during the previous summer; but now her menses had been absent for the last five months, her abdomen had gradually enlarged, and for the last month she felt the movements of a child *in utero* precisely as in former pregnancies. The signs in this condition, however, by any change in the breasts were wanting; and the morning sickness, which always troubled her excessively when she was carrying each of her children, was absent. Her general health was excellent, and she had been latterly increasing in weight.

On examination, the uterus was found to be undeveloped and not affected with any disease.

There was evidently a considerable increase of fat in the abdominal walls; but, aside from this, no cause could be clearly discovered for distension.

Case II.—A. H., aged 34, who was married, and had had six children and one abortion, sought advice at the clinique for a pregnancy, as she thought, which had prolonged itself beyond the normal period. Thirteen months ago her courses stopped for two months, but subsequently there has been regularly a very slight red stain. She has had morning sickness and feelings similar to those she experienced with all of her children; but there is no change in the breasts; they are undeveloped, and the areolæ and follicles are unchanged.

About three months after the cessation of the courses she discovered a tumor in the left iliac region that was movable, changing its position as she lay on one side or the other. This tumor seemed to her to increase in size continuously, and at present her abdomen is distended as though with a nine month's fœtus. Shortly after the time above mentioned she felt life, and continued to do so, precisely as in other pregnancies, to the period when she should have been confined. Now, in a disturbance with a drunken man, she was thrown violently and struck on her side against a trunk, with a force so great that a blood-mark, the size of the open hand, was formed. After this injury the motions of the child became more and more feeble, and eventually ceased altogether in two weeks' time. Chills several times a day, coldness of the extremities, difficulty in evacuating the bladder, and dizzy, swimming sensations, were now experienced. On examination, no enlargement of the womb, morbid growth, or other diseased condition could be detected, only a distension of the abdomen, from the bowels, which were very torpid and filled with gas and fæces. There was a considerable deposition of fat in the cellular tissue; indeed the woman was corpulent and much above the average weight, yet she was markedly deficient in red blood, and was now as the investigation showed, suffering from the symptoms of anæmia, which either originated the defective menstruation or arose from it. My experience teaches me that there may be a poverty in the red globules without its necessarily rendering the individual thin and spare, and that often there is defect in persons grossly fat; also, that anæmia is frequently the cause of menstrual irregularities, and, *vice versa*, amenorrhœa is very many times the cause of anæmia.

The diagnosis in this case was scanty menstruation, which was supposed to be the occasion of the other morbid conditions.

Case III.—Several years ago a woman called at my office to engage my services in her confinement, which was expected to come off in a couple of weeks. As on inquiry she lacked some of the more ordinary signs of pregnancy, I instituted a tactile examination, when the uterus was discovered to be undeveloped. Her disappointment was great at the blighting of her hopes. The particulars of this case I am unable to give, since by neglect I omitted making a record at the time; and I can recall nothing in regard to it, excepting that she had been married about a year and was thirty or more years of age.

Case IV.—In April, 1862, a married woman, aged 28, the mother of four children, and the subject of two abortions, presented herself at the clinique. She supposed herself near her time, as she was greatly increased in size, and had felt life for four and a half months. She applied on account of a severe flooding which had seized her three times. On examination, the uterus was found undeveloped and retroflected.

Case V.—During the past summer, my friend Dr. J. E. Clark, requested me to examine a lady who thought she had more than completed the period of utero-gestation. She had been married rather more than a year, and notwitstanding her courses were regular though scanty, she had for ten months been going through the phases of pregnancy, and experiencing the phenomena usually attending it, even to feeling the fœtal movements. She had prepared everything for the hoped-for event, and now she suffered the preliminary but irregular pains of labor, that, starting from the lumbar region, passed forward and downward through the pelvis. She was very corpulent, her breasts were very large, but lacking the signs of gestation, and her abdomen was much increased in size and resonant. On examination, the uterus was found to be of the virgin size. The illusion of the lady was banished by aloes and assafœtida, which unloaded the bowels and removed the flatus.

Case VI.—On the twenty-seventh of last August I was called at eleven o'clock, p. m., to see a woman at the Station House in Court street. On my arrival, I found the captain of the police in attendance, who told me the woman was in labor, and that the waters had come away. I found she had severe pains about every five to ten minutes, like those of labor, which were attended with an expulsive effort, as we see in the second stage. It seems that she had had in the early part of the evening hysterical convulsions in Montague street, and was taken into a house by a lady. Subsequently she tried to make her way home, but was obliged to call a policeman to her assistance.

The examination revealing an undeveloped uterus, she was sent to the hospital, where, on the next morning, I obtained the following history:—A. K., æt. 17 years, had only menstruated twice before her marriage, which took place ten months ago; but subsequently, for four "turns," she was "unwell" regularly and naturally. For the last six "periods" her "courses" have failed, excepting once four weeks ago. Until the suppression, her health was perfect, but afterwards she had nausea in the morning, sour stomach, etc.; but as the nausea went off, as it did in an hour or two, she felt well the rest of the day, and had a good appetite for dinner and supper. She had frequent, sometimes difficult, urination; a feeling of weight and pressure down the pelvis, particularly on walking; a sense of weakness through the back and hips, and a white vaginal discharge, but no pruritus, burning or scalding sensations. Her bowels operated mostly every day, and her abdomen was swollen and felt tender generally, but was more particularly sensitive over the stomach and edge of the liver. Her size, augmenting gradually for the last six months, has obliged her to let out her dresses. On examination, there is found no change in the breasts, no growth in the abdomen, and no development of the uterus. At the third day of her stay in the hospital, after being questioned in regard to fœtal movements, she felt life, and continued to do so when she was dismissed. Two weeks subsequently, when she called at my office, she still persisted obstinately in the opinion that she was pregnant, and my professional dictum had no weight with her perverted and deluded imagination. The treatment consisted of cathartic doses of blue mass and aloes, which, bringing away retained fæces and flatus, reduced her size promptly, removed the abdominal tenderness, and corrected the gastric and hepatic secretions.

Case VII.—In September last, M. A., a married woman, forty years of age, who never conceived unless now pregnant, came to the clinique to learn what might be her prospects touching family matters. Unfortunately, four years ago her hopes were blighted when everything—a gradual enlargement of the abdomen to the nine months' standard, the development of the breasts, which secreted a milky fluid, and the movements of the child, which were felt for four months—seemed promising. Gradually the swelling disappeared, without any notable evacuation either of fæces or gas. During this quasi-gestation she was healthy, had a good appetite, and a regular free state of the menstrual function. She now states that she began two months ago to enlarge again, and that she has the same symptoms as before, excepting the fœtal movements and the secretion of milk. Her menses are regular and free, and were neither now nor on the former occasion interrupted.

On examination, silver lines were found on the abdominal walls, and flatus in the large intestines; but the uterus was of its normal, unimpregnated size. The treatment was the same as in the fifth case.

Case VIII.—Mrs. A. L., aged 36, married, the mother of two children, and the subject of one miscarriage, came to the clinique January last. Five months ago she was delivered of a false conception—a fleshy mass, with no appearance of organization, the size of the two closed fists. Excepting the stoppage of the menses for a period of four months, she had had no symptoms of pregnancy, and no feelings such as she formerly experienced when in the family-way. Her condition was one of debility and weakness, which were further increased by a profuse flooding that attended the discharge of the false conception. Her present symptoms are morning-sickness, weakness in the last lumbar vertebræ, tenderness in the right iliac fossa, leucorrhœa, a greatly increased size of the abdomen, and an absence of the courses for the last four months, excepting a slight red stain three days since. She is tender over the margin of the liver, exhibits signs of hepatic torpor, and disorder of the gastric functions. She has not felt life, and experiences no darting pains through her breasts, which are unchanged in their size and in the color of their areolæ, though warmer than the surrounding parts and nodulated from the enlarged milk tubes.

On examination, the uterus was found to be of its natural size and free from any disease, and the abdomen to be very resonant.

She was ordered the following prescription: R. Hydrarg. chlorid. mit. gr. viij.; resinæ jalapæ gr. ij.; sacch. albi ʒ ss.; M. This was to be followed by castor oil in case it failed to operate. The evacuations by this cathartic were very dark, but there were no evidences of the bowels being distended by fæces, and no wind was passed, although it was found on examining her again that the gas had left the intestines, and that the prominence of the abdomen had subsided. The calomel seemed to break up the chain of morbid sympathies and restore the digestive functions to their normal condition.

Case IX.—Mrs. L. M., æt. 24, and married eight years, has during the last four years miscarried three times, but she never had a living child. She had been treated by Dr. C. by the speculum for six months without obtaining any benefit. For the last six months her menses, though regular and without pain, are extremely scanty—a mere red stain for one day. She has no leucorrhœa, urinary trouble, or itching, scalding, or forcing sensations in the pelvis, but suffers from great tenderness in the left iliac fossa, and a severe pain in the sacrum and last lumbar vertebræ that extends over the left ilium and down the left leg posteriorly. This pain is increased by exercise, and relieved by lying down. She is slightly constipated, but her stomach is not disordered, and she takes her food with a relish. She has symptoms of pregnancy, shooting pains in the breasts, enlarged milk tubes, distinct follicles, and a light brown color of the areola. Six months previously she suffered severely from morning sickness, and had fainting fits, to which she is always subject during gestation.

By the touch the uterus was found prolapsed to the perinæum, lying nearly in the axis of the excavation, excepting that the fundus was more backward and pressed under the promontory of the sacrum. The uterus could be readily elevated on the point of the finger, was not enlarged or congested, and its neck was not thrown forward.

By the speculum the cervix was found to be free from disease.

This patient, for a month and a half, took internally the pyrophosphate of iron, and used vaginal injections of alum; at the end of which time she felt very badly, and was obliged, from the intensity of the pelvic pains, to keep her bed most of the day. She had menstruated twice very freely, a week each period, and with great pain, The areolæ were of a deeper brown than at first.

The prescriptions were renewed, and on the following week a globe-pessary was introduced.

My notes state that she returned at the end of a month, felt much better, and was relieved of her troublesome symptoms by the use of the instrument. Whether the benefit was permanent is not known.

Case X.—E. L., æt. 30 years, the mother of two children, has the following symptoms:—Menses every third week, free, and lasting from six to eight days; bowels regular; appetite variable; tenderness over the stomach on pressure; no gastric disorder, no hysteria, and no pelvic irritation or suffering. Her husband left for the war in January, 1863, and in the last part of April following she felt movements in the abdomen. Four and a half months after feeling these movements, her size was equal to that of a woman at the ninth month, and her breasts were distended with milk. When the supposed full term had arrived, she was taken with pains, which lasted for three days, and were violent enough to cause her to seize hold of some object to steady and support herself. After this she had no more pains, the distension of her abdomen gradually subsided, her breasts grew smaller, and the milk dried up. In January, 1864, the symptoms of pregnancy had disappeared. In June following, when I first saw her, she was suffering from nervous prostration and gastric disorder. She was ordered resinous purgatives and iron. Whether her health was completely restored is not known.

*Commentary.*—The subject of spurious pregnancy, though of high practical import, has not received that attention from systematic writers which its obscurity would seem to demand. In truth it is scarcely alluded to, except incidentally, when the signs of pregnancy are discussed; and never with a completeness sufficient to aid the general practitioner in diagnosticating such cases, or understanding the phenomena they present. The few instances recorded in the medical journals stand as isolated facts—rare and curious facts—but no one thinks they are other than exceptional, or that the morbid condition can be studied to advantage or explained satisfactorily. Hence a mystery hangs over this disease and shrouds its causation in an obscurity so great that well educated physicians frequently commit the most grievous blunders, which, perhaps, are only rectified at the commencement of a spurious labor, when an empty womb is determined by the touch.

*Diagnosis.*—The diagnosis the first three months is well-nigh impossible, since a congestive state of the uterus and its appendages will occasion the same sympathetic phenomena as a genuine pregnancy—enlargement and shooting pains in the breasts, changes in the areolæ and their follicles, etc.—but there is this difference, disease produces an unnatural, pregnancy a physiological congestion of the uterus; in the former the general health will deteriorate and be gradually undermined; in the latter it will remain intact, or, if disturbed at first, will subsequently improve, as is shown by a good appetite, vigorous digestion, increase in flesh, and a full, firm pulse. In almost any case, when the menses have failed, should we have evidences from the pulse, the desire for and the proper assimilation of food, that the organic functions are carried on with regularity and in perfection, we may be tolerably certain that impregnation has taken place; since a morbid condition of the uterine organs would specially involve the ganglionic nervous system, and perturb or subvert every function over which it presides. When gestation has advanced three and a half to four months, the line of demarcation between the morbid and the physiological becomes more clearly defined, the breast-signs are better pronounced, and the enlargement and gradual growth of the uterus are very apparent to the touch. When the indications of gestation above mentioned increase steadily for some weeks, you may form all but a positive diagnosis.

From four to five, and five to six months, no practitioner is excusable in not being able to give a proper answer, and deciding positively the existince or non-existence of impregnation; since now, in addition to the more characteristic changes in the breasts and the presence of the bulky uterine globe that is detected by palpitation and the touch, or both united, the movements of the child, perceptible to the mother and attendant, balottement and ausculation will determine beyond a peradventure the fact of conception. Nevertheless, the most experienced accoucheurs, at this, or even as late as the full period, have at times been at fault, being at the outset misled by the spurious symptoms of pregnancy, and finally confirmed in the error by the patient asserting that she feels the movements of the child.

Professor Bedford relates a case, in his work on Obstetrics, of a lady suffering with ascites, so strenuously positive as to the reality of her pregnancy, of which she was certain from the strong movements of the child, that she frequently wrung from him an equivocal but reluctant assent that he also felt these movements. He was thus led on, without making an examination, to the completion of the nine months, when, false labor pains setting in, the uterus was found undeveloped. The lady died four days subsequently.

Dr. Keiller (Monthly Journal, and Braithwaite, No. xxii, page 313) relates a case of spurious pregnancy to which he was called by the attending physician to perform the Cæsarean section by reason of the very painful and protracted nature of the labor, which had reduced the patient to the verge of exhaustion. The motions of the child could be felt and seen distinctly, and were thought by the patient to be so violent that it seemed as if the child "would leap through her side." Professor Simpson, in his recent work on the Diseases of Women, relates a great many instances of this morbid condition; but his apparent desire to make much of his subject, his statement that virgins may have the disease, as had been observed in the case of sluts excluded from the society of the male at the time of "heat," his assertion that spurious imitates actual pregnancy so perfectly as to be its exact counterpart, and his grouping under this head all cases where a woman suspects or fears that she is pregnant, form such a jumbled, distorted and colored picture, that his descriptions are devoid of practical value, and involve us in greater doubt and hesitation than the silence of our obstetrical writers. The truth is, that a spurious pregnancy like those that I have related, is only of occasional occurrence, and can be diagnosticated usually without much difficulty.

*Cause.*—The cause appears to be an irritation of the uterine organs, not from morbid growth, congestion, inflammation, or any other pathological state open to the investigation of our senses; but from a defect in, or absence of, the menstrual function, from an irritability caused by excessive coitus in the newly married, from the change of life, or from displacement of the uterus, as we observe in partial retroversion or retroflexion. When the menses are scanty or absent for some months, we almost invariably find the bowels torpid, flatulent and distended, the stomach nauseated, filled wi h morbid secretions and loathing food, and the liver overcharged with its secretion, as is evinced by the dusky, yellow state of the skin. The nervous system is seriously implicated, as the whole family of the neuroses testify, and seems to be imperfectly sustained in its due balance by a defective blood. At this point the mind loses its healthful tone and may become the prey to any illusion. Should the female desire offspring very much, she will very likely brood over her disappointed hopes, and eventually become a monomaniac on this subject. In my opinion, the menstrual fluid is both a hemorrhage and an excretion, and does, like the bile, eliminate from the blood certain noxious elements which, if retained, disorder the circulation and perturb the nervous system. Probably most cases of pseudo-pregnancy may be referred to an imperfect functional activity of the uterus, and the perverted state of mind thence arising. The remainder arise from some obscure irritation of the generative organs. The condition has some analogy to hysteria in the torpid, distended, and flatulent state of the bowels, the unbalanced mind and the perversion of the nerve centres, and doubtless is one of the protean forms of this disease. As the abdominal muscles are put upon the stretch, and are consequently fatigued and weakened, some of their fibres may contract irregularly and spasmodically, and thus imitate the movements of a child in utero.

*Treatment.*—The treatment of spurious pregnancy is sufficiently indicated by the cases related above. A pre-requisite to a satisfactory management of any case is a positive diagnosis, so clearly made out that we can unhesitatingly dispel the woman's false hopes. The illusion must be banished before we can hope to combat successfully the functional disturbances that attend in its train. Of these disturbances, the most marked are torpor of the liver and atony of the muscular coats of the digestive canal. Purgatives of the resinous kind, with or without mercurials, according to the state of the biliary secretion, will be demanded in most instances for the restoration of the stomach, bowels and liver, to a proper discharge of their offices. Occasionally it will be necessary for the expulsion of flatus to make the cathartic medicine stimulating and carminative by the addition of asafœtida or turpentine. As the muscular coats of the intestines have, by distension, lost their tonicity, we must re-awaken excitability and contractility by local stimulation, and then continue this impression for some days; but when a proper action of the bowels is renewed, this must be eventually sustained by laxatives, or preferably by vegetable articles of diet. Order having been restored in "the storehouse and shop of the whole body," the elements of nutrition will be presented to the blood, which, if defective, may now find the materials for its reparation. Should the red globules be deficient, we may now resort with advantage to some of the many preparations of iron. As the blood is renewed the nervous system will feel the quickening influence, animal force and vigor will radiate to every part of the system, and the generative organs will be restored to their normal condition. Thus menstrual irregularities or deficiencies will be remedied, and the immediate cause, frequently, of the patient's infatuation will be removed. If displacement of the uterus, by irritation of the organic nervous system, conspires to the production of the spurious symptoms of pregnancy, the use of pessaries, or other means, will be necessary before a permanent cure can be effected. In fine, we must banish the hallucinations of the patient, and then correct the functional disorders, whatever they may be, that have arisen as results.

I will in this connection relate a case of nymphomania that came under my notice in June, 1862, that seemed to arise from a premature cessation of the "courses." A. M., aged 44, and single, has had her menses suppressed for ten years, with the exception of a few months four years ago, and one "turn" two months back, when she had a considerable "show." She was brought to the clinique by a female friend. She was moping, spiritless, gloomy, and greatly distressed in mind, and had fled the house of her employer, where she had lived many years, for fear she should commit some overt act, some great indiscretion, with her master's son, a boy eighteen years of age. She stated, without any reserve or apparent violence to her delicacy, that she had the most uncontrollable passion for this young lad, could not bear to have him a moment from her sight, and had a burning desire to sleep with him. She struggled against these feelings, being, according to the statement of her friend, a person of rigid morals, and one having a high sense of religious duty, but eventually the infatuation became so complete and irresistable that she suddenly took safety from her temptation in flight. The patient was corpulent and full-blooded, had a wild, nervous excitability about her, would not eat for fear of poisoning, and suffered from gastric, hepatic, and intestinal disorder; yet she was not entirely bereft of reason, as was evident from her leaving her master's house to avoid temptation, and her ready assent to an examination for uterine disease, which I thought might be the cause of her present state. The examination revealed the hymen intact, but failed to disclose any disease of the uterus or vagina. This patient was sent to the Lunatic Asylum at Flatbush, and nothing further of her history has come to my knowledge. To me it seems clear that the symptoms in the case arose from the amenorrhœa, and that the patient's blood was poisoned by the retension of certain elements that should have been eliminated by the menstrual fluid, whence originated this peculiar manifestation of hysteria. Her real condition was not far removed from that other monomaniacal class—the subjects of an imaginary pregnancy.

Dr. Hutchison referred to the case of a lady, whom he saw, when she supposed herself five months pregnant. The *menses* were scanty, the *abdomen* enlarged, the *areola* discolored, and as she affirmed, fœtal movements were distinctly felt. He could not detect the pulsations of the fœtal heart, yet he gave it as his opinion that she was pregnant. He watched her until the ninth month, when she suffered from severe pains; a proper discharge of blood occurred, and a *gush* of wind proceeded from the uterus. In conclusion the Doctor thought that *good* resulted, as well from recounting the mistake, as the successes of Physicians.

Dr. Burge saw a lady, who had all the signs of pregnancy, and enjoyed good health. She continued to manifest such symptoms for eighteen months, when she was delivered of a fine child.

Dr. Hart had been engaged, at one time, to attend a woman who, after the lapse of ten months, and no signs of labor coming on, was examined by him, and the *abdomen* found to be flaccid, and the *uterus* undeveloped.

Dr. Enos remarked that he was sent for, some time ago, to attend a patient who supposed herself in labor. Upon an examination, however, he found the uterus undeveloped. The Doctor also mentioned a case, he saw in the country, of a woman, whose abdomen was enlarged, a *motion* distinct to the patient, and also to the physician. He had never seen the motions of a fœtus more distinct; yet the abdomen was resonant, and the uterus undeveloped, tumor being found.

